# Granulocyte Colony-Stimulating Factor Promotes Atherosclerosis in High-Fat Diet Rabbits

**DOI:** 10.3390/ijms14034805

**Published:** 2013-02-28

**Authors:** Zhaohui Hu, Jie Zhang, Aili Guan, Hui Gong, Ming Yang, Guoping Zhang, Jianguo Jia, Hong Ma, Chunjie Yang, Junbo Ge, Yunzeng Zou

**Affiliations:** 1 Shanghai Institute of Cardiovascular Diseases, Zhongshan Hospital and Institutes of Biomedical Sciences, Fudan University, Shanghai 200032, China; E-Mails: zhaohuihu2006@yahoo.com.cn (Z.H.); alguansh912@yahoo.com.cn (A.G.); gonghui2005@fudan.edu.cn (H.G.); yang.ming@zs-hopital.sh.cn (M.Y.); gpzhang@yahoo.com.cn (G.Z.); jianguo_jia@yahoo.com.cn (J.J.); yang.chunjie@zs-hospital.sh.cn (C.Y.); ge.junbo@zs-hospital.sh.cn (J.G.); 2 Department of Radiology, Shanghai First People’s Hospital, School of Medicine, Shanghai Jiaotong University, Shanghai 200080, China; E-Mail: zhangjie1975@163.com; 3 Department of Cardiology, the Second Affiliated Hospital, College of Medicine, Zhejiang University, Hangzhou 310009, China; E-Mail: mahongchn@gmail.com

**Keywords:** atherosclerosis, endothelial cell, ET-1, G-CSF, apoptosis, hyperlipidemia

## Abstract

Granulocyte-colony stimulating factor (G-CSF) has been reported to improve the function of infarcted heart, but its effects on atherosclerosis are unclear. Here we examined the effects and the potential mechanisms in the high-fat diet rabbit model. Six-month-old male New Zealand white rabbits, fed a high-cholesterol diet or a normal diet for 10 weeks, were treated with vehicle or G-CSF. G-CSF increased lesion area in the thoracic aorta and the plasma levels of total cholesterol (TC) and low-density lipoprotein-cholesterol (LDL-C) at the early phase in the high-fat diet group. High-fat diet-induced arterial endothelium damage and apoptosis were greatly aggravated by G-CSF treatment. *In vivo*, G-CSF impaired apoptosis induced by oxidized low density lipoprotein (OX-LDL) but it had little effect on cultured endothelial cells (ECs) with vehicle treatment. Further research revealed that G-CSF promoted the upregulation of endothelin-1 (ET-1) and the downregulation of endothelial nitric oxide synthase (eNOS) of thoracic aortae induced by a high-fat diet. *In vitro*, the effects of G-CSF on expression of ET-1 and eNOS in cultured ECs were consistent with those *in vivo*. Our results suggested that G-CSF exacerbates lipid abnormity and endothelium damage in hyperlipidemia rabbits, thereby resulting in the deterioration of atherosclerosis and that the ET-1/eNOS system may regulate the progression.

## 1. Introduction

Granulocyte-colony stimulating factor (G-CSF) has been shown to repair the infarcted heart not only by inducing mobilization of bone marrow stem cells [1] but also by directly acting on cardiomyocytes [2]. However, there are also reports indicating that G-CSF administration increased the incidence of coronary re-stenosis or progression of coronary lesions in patients with myocardial infarction (MI) [3] although another study obtained negative results [4]. Clinical concern about patients with MI is that they have, more or less, attenuation of the coronary arteries as a consequence of progression of atherosclerosis [5]. The use of G-CSF for patients with atherosclerosis has attracted much attention. The feasibility, safety, and efficacy of G-CSF therapy for cardiac repair therefore require further investigation. Although it is well known that hyperlipidemia is one of the most important risk factors for the development of atherosclerosis [6], only a few patients with coronary artery disease (CAD) are accompanied by hyperlipidemia. We therefore suppose that the different results of clinical studies on G-CSF administration are due to differences in plasma lipid levels of MI patients.

In this study, to elucidate whether the effects of G-CSF on the development of atherosclerosis are different between subjects with or without hyperlipidemia, we used rabbit models, fed a normal or high-fat diet and examined the atherosclerosis in the thoracic aortae of these animals with or without G-CSF treatment.

## 2. Results and Discussion

**Figure 1:**
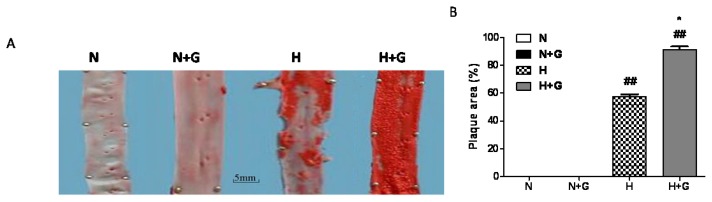
The effect of granulocyte-colony stimulating factor (G-CSF) on atherosclerosis in thoracic aortae. (**A**) Representative photographs of Sudan III-stained thoracic aortae; (**B**) Quantitative analysis of the plaque area in thoracic aortae. Plaque area was evaluated as the percentage of lesion area of the total surface area of intima. A, Scale bar: 5 mm. N: normal diet group, N + G: normal diet group treated with G-CSF, H: high-fat diet group, H + G. high-fat diet group treated with G-CSF. Results were expressed as means ± SEM. *N* ≥ 3. *p < 0.05, normal diet +G-CSF *vs.* normal diet group, or high-fat diet +G-CSF *vs.* high-fat diet group; ^##^*p* < 0.01 high-fat diet group *vs.* normal diet group or high-fat diet +G-CSF *vs.* normal diet +G-CSF.

G-CSF has been reported to improve the function of infarcted heart, but its effects on atherosclerosis are unclear. Here, our results show that G-CSF promotes atherosclerosis in rabbits with a high-fat diet. The inner surfaces of the aortae of the normal diet group with or without G-CSF treatment were smooth and flat, and showed no positive Sudan III staining (Figure 1A). In the high-fat-fed group, obvious atherosclerotic loci were observed in the thoracic aorta. In high-fat diet plus G-CSF treated rabbits, the luminal surfaces of the aortas were more deeply stained with Sudan III corresponding to severe atherosclerotic changes (Figure 1A). G-CSF greatly increased the area of lipid plaque in the thoracic aortae in the high-fat diet group but it had little effect on the inner surface of the aortas in the normal diet group (Figure 1A,B). Therefore, our present data showed that G-CSF promoted the progression of atherosclerosis in the high-fat diet.

However, G-CSF has been observed to prevent the progression of atherosclerosis in MI and balloon injury rabbits in the study of Hasegawa H [7]. The protective outcome may be related to the effect of G-CSF on mobilizing progenitor cells to repair the seriously damaged vessels. Takai, H. *et al.* recently reported that G-CSF had no adverse effects on atherosclerotic lesions in high cholesterol-fed miniature swine [8], the different results may be due to the difference in the dose and the animal model of G-CSF treatment. The findings of Haghighat support our results in that G-CSF exacerbates atherosclerosis in apolipoprotein e-deficient mice, but the mechanisms were unclear [9].

**Figure 2:**
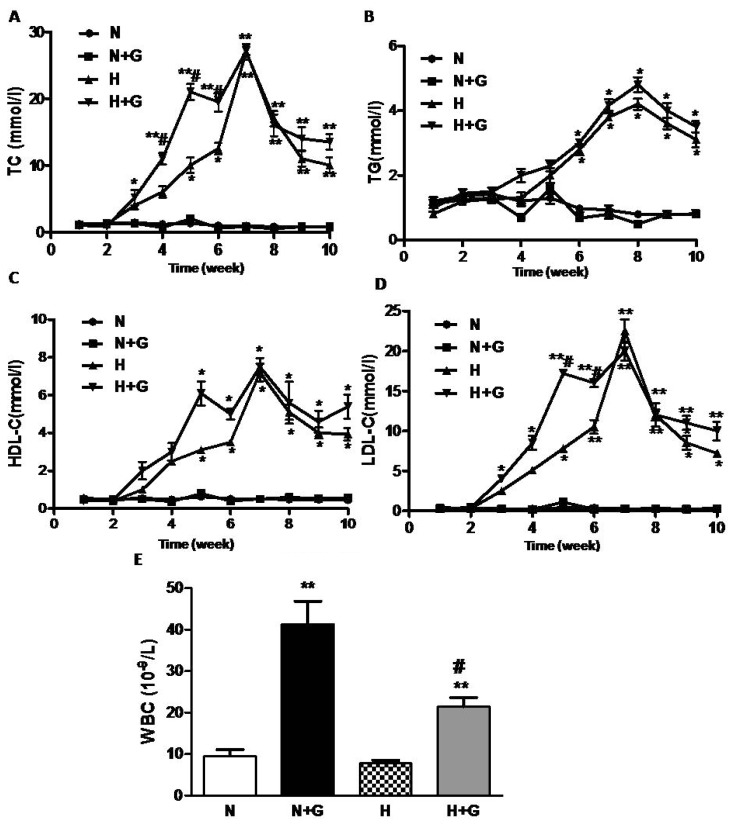
The changes of plasma lipids and the white blood cells (WBC) counts. (**A**) total cholesterol (TC) (mmol/L) (**B**) triglyceride (TG) (mmol/L) (**C**) low density lipoprotein-cholesterol (LDL-C) (mmol/L) (**D**) high cholesterol density lipoprotein-cholesterol (HDL-C) (mmol/L) N: normal diet group (*n* = 5). (**E**) the WBC counts in blood. N + G: normal diet group treated with G-CSF (*n* = 6), H: high-fat diet group (*n* = 6). H + G: high-fat diet group treated with G-CSF (*n* = 7). Results are expressed as means ± SEM. *p < 0.05, **p < 0.01, normal diet +G-CSF *vs.* normal diet group, or high-fat diet +G-CSF *vs.* high-fat diet group; ^#^*p* < 0.05 high-fat diet group *vs.* normal diet group or high-fat diet +G-CSF *vs.* normal diet +G-CSF. Total cholesterol (TC), triglyceride (TG), (LDL-C) and (HDL-C).

Hyperlipidemia is well recognized as an important risk factor for atherosclerosis [6], and hypercholesterolemic atherosclerosis has been associated with an increase in the serum TG, TC, LDL-C, and the ratio of TC/HDL-C [10,11]. We detected the levels of plasma lipid per week during the whole study. TC, LDL-C, TG and HDL-C in plasma remained unchanged in the rabbits fed normal diet, but they increased at week 5–6 and peaked at week 7 after being fed a high-fat diet (Figure 2A–D). G-CSF injection did not affect the levels of TC, LDL-C, TG and HDL-C in rabbits fed a normal diet (Figure 2A–C). In the high-fat diet-fed group, G-CSF treatment advanced the increase in TC and LDL-C from week 5–6 to week 3 but it did not increase the peak value and did not advance the peak time of TC and LDL-C (Figure 2A,C). TG and HDL-C increased from week 5–6 and remained at high levels in rabbits after being fed the high-fat diet. However, the levels of TG and HDL-C showed no difference between G-CSF and vehicle treatment in the high-fat diet group (Figure 2B,D).

In the present study, G-CSF did not affect the plasma lipids in the normal diet group, but it increased plasma TC and LDL-C in the high-fat diet groups at the early phase although it had little effect on the peak concentration of plasma lipid. Haghighat reported that G-CSF did not affect serum lipids in apolipoprotein e-deficient mice [9]. However, they detected serum lipids only at the end of the experiment. Many studies have found LDL-C to be the most dangerous among the serum lipids. Oxidized LDL particles (and certain other modified forms of LDL) are ligands for “scavenger” receptors on macrophages and can therefore convert them to cholesterol-loaded foam cells, characteristic of the earliest atherosclerotic lesions, the fatty streaks [5,12–14] which are involved in the initiation and promotion of atherosclerosis [15]. Moreover, in animal experiments, the LDL of hypercholesterolemic rabbits was more susceptible to oxidative modification than that of normolipidemic rabbits [6]. So G-CSF-induced the early increase of plasma lipids (especially LDL-C) and may accelerate plaque formation in rabbits with a high-fat diet. In the G-CSF-treated groups, a significant increase inWBC counts was observed in both normal diet and high-fat diet group (Figure 1E). However, the increase in WBC number in the blood by G-CSF treatment was less in high-fat diet rabbits than in the normal diet group. High-fat diet did not affect the value of WBC. These results were consistent with a previous study [8], but the effect of G-CSF on WBC counts in the high-fat diet group was not observed. However, the major pharmacological activity of G-CSF could not explain the worsening effect of G-CSF on atheroscerlosis.

**Figure 3:**
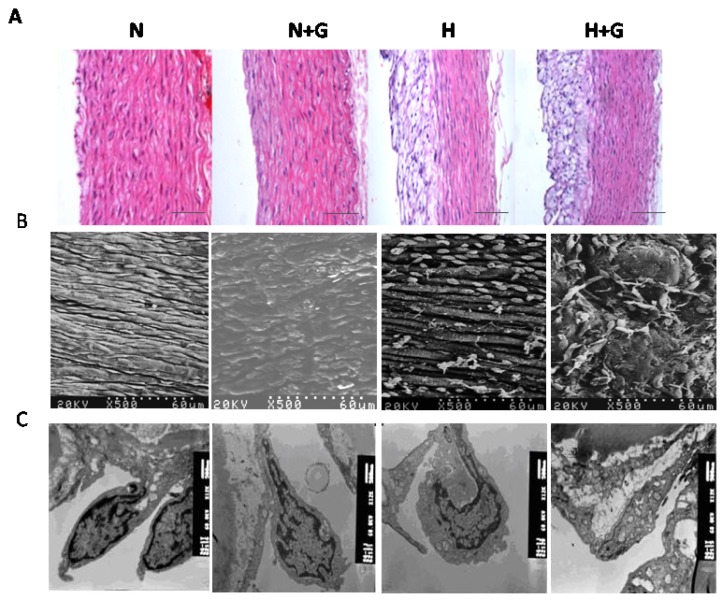
Impairment and apoptosis analysis of endothelial cells of thoracic aortae. (**A**) HE-stained thoracic aortae were analyzed by optical microscope. Ultrastructural changes of endothelial cells of thoracic aortae were observed by scanning electron microscope (**B**) and transmission electron microscope (**C**) analysis. N: normal diet group (*n* = 3). N + G: normal diet group treated with G-CSF (*n* = 3). H: high-fat diet group (*n* = 3). H + G: high-fat diet group treated with G-CSF (*n* = 3). (**A**): Scale bar: 200 μm.

Vascular endothelial cell (VEC) injury represents a major initiating step in the process of atherosclerosis. Normal endothelium is antithrombogenic, and if injured, it promotes platelet aggregation and coagulation. Endothelial damage can cause vasospasm, intimal hyperplasia, and arteriosclerotic acceleration [16]. In our study, obvious plaque can be observed in the thoracic aortas in high-fat diet rabbits, but not in normal diet groups. G-CSF greatly increased the atherosclerotic lesion area in the thoracic aortas of high-fat diet rabbits. HE staining showed G-CSF did not influence the intimal structure in the normal diet group. In the high-fat diet group, the intima of the thoracic aorta was composed of a thin, fibromuscular layer covered by a large, foam cell-rich outermost layer, G-CSF had little effect on it (Figure 3A). We next performed electron microscope analysis to examine the ultrastructural change of endothelial cells (ECs) in the thoracic aorta. Scanning electron microscope analysis showed that ECs of the thoracic aorta in the normal diet group were arranged orderly and joined by tight junctions (Figure 3B). G-CSF treatment only induced slightly disordered arrangement in ECs (Figure 3B). High-fat diet resulted in obvious plaque as well as loose and irregular arrangement of ECs. Deformation, missing and apoptosis of the ECs, lipid droplet-like changes, cell debris and a few scattered erythrocyte adhering to the endothelial monolayer,r could also be observed (Figure 3B). The EC’s apoptosis and lipid droplet-like changes were the most serious in the high-fat diet group treated with G-CSF among all the groups (Figure 3B). Ultrastructural intimal changes of the thoracic aorta were observed by transmission electron microscope. In the normal diet group, ECs of the thoracic aorta showed normal structure, and complete inter-endothelial contacts. G-CSF treatment in normal diet rabbits only induced a loose arrangement of ECs, expansion of the endoplasmic reticulum and slight mitochondria swelling (Figure 3C). In rabbits fed high-fat diet, ECs containing lipid droplets were more loosely arranged. Inter-endothelial contacts were incomplete or completely absent. EC’s morphology was seriously destroyed, characterized by blebbing, and spike-like protrusions of the cell membrane, loss of boundary of cytoplasm and mitochondrial swelling (Figure 3C). However, G-CSF plus high-fat diet incurred the gravest damage in ECs, including numerous lipid droplets, intracytoplasmic vacuoles, basement membrane detachment and lysis of mitochondria (Figure 3C). These results showed that G-CSF induced slight damage in ECs in the thoracic aorta of the normal diet group, but it significantly promoted damage and apoptosis in ECs induced by the high-fat diet.

**Figure 4:**
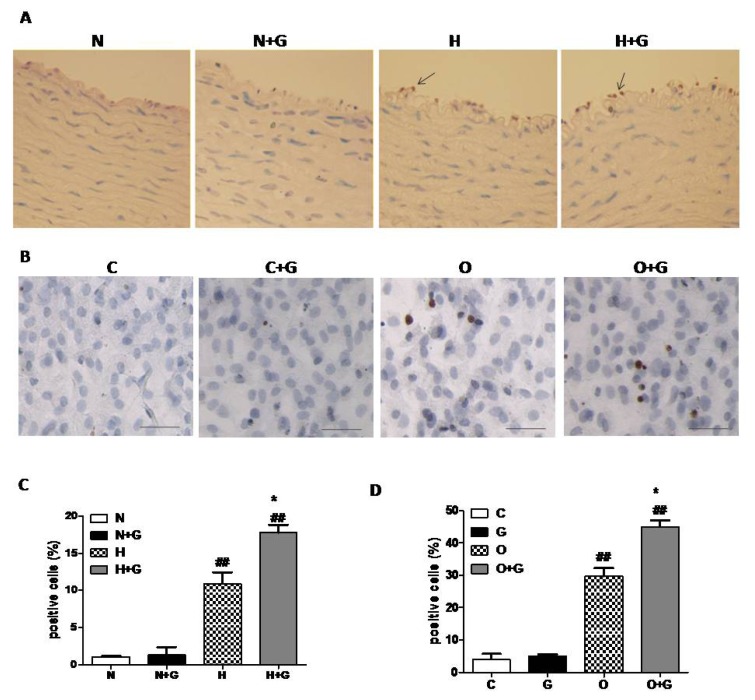
The effect of G-CSF on apoptosis of endothelial cells in *vivo* and *in vitro*. TUNEL analysis of thoracic aortae (**A**) non-plaque-area) and cultured endothelial cells (**B**) N: normal diet group (*n* = 3). N + G: normal diet group treated with G-CSF (*n* = 3). H: high-fat diet group (*n* = 3). H + G: high-fat diet group treated with G-CSF (*n* = 3). *p < 0.05, **p < 0.01, normal diet +G-CSF *vs.* normal diet group, or high-fat diet +G-CSF *vs.* high-fat diet group ^#^*p* < 0.05 ^##^*p* < 0.01 high-fat diet group *vs.* normal diet group or high-fat diet +G-CSF *vs.* normal diet + G-CSF. The cultured endothelial cells were incubated with G-CSF (50 ng/mL) or vehicle for 30 min followed by OX-LDL (50 μg/mL) or vehicle for 24 h. The percentage of TUNEL positive nuclei were calculated in the thoracic aorta (**C**) and cultured endothelial cells (**D**). C: Control group. G: G-CSF group. O: OX-LDL group. O + G: OX-LDL + G-CSF group. Scale bar: 50 μm. Results are expressed as means ± SEM. *p < 0.05, G-CSF *vs.* control group, or OX-LDL +G-CSF *vs.* OX-LDL group ^##^*p* < 0.01 OX-LDL *vs.* control group or OX-LDL + G-CSF *vs.* G-CSF group. Each experiment was repeated independently at least three times. (**A**) Scale bar: 50 μm. (**B**) Scale bar: 200 μm.

In order to examine whether G-CSF exerts a synergestic effect on apoptosis in endothelial cells, we next performed TUNEL analysis on thoracic aorta tissue. In the lesion area of the thoracic aorta, ECs were seriously damaged, so we analyzed the data in the non-lesion area. The results revealed that G-CSF significantly increased TUNEL-positive ECs in the non-plaque area of the thoracic aorta in high-fat diet rabbits, but it did not induce apoptosis of ECs in normal diet rabbits (Figure 4A,C). We next evaluated the effect of G-CSF on apoptosis of cultured ECs treated with OX-LDL to confirm the result. ECs from thoracic aortae of one week old rabbits were cultured and incubated with OX-LDL (50 μg/mL) or vehicle for 24 h with or without G-CSF (50 ng/mL, 30 min) pretreatment. TUNEL analysis indicated that G-CSF greatly increased the number of apoptotic cells treated with OX-LDL, but it did not induce apoptosis in the ECs without OX-LDL treatment (Figure 4B,D). These data indicated that G-CSF exacerbates apoptosis of ECs induced by high-fat diet *in vitro* and *in vivo* which may result in a worsening effect on atherosclerosis.

**Figure 5:**
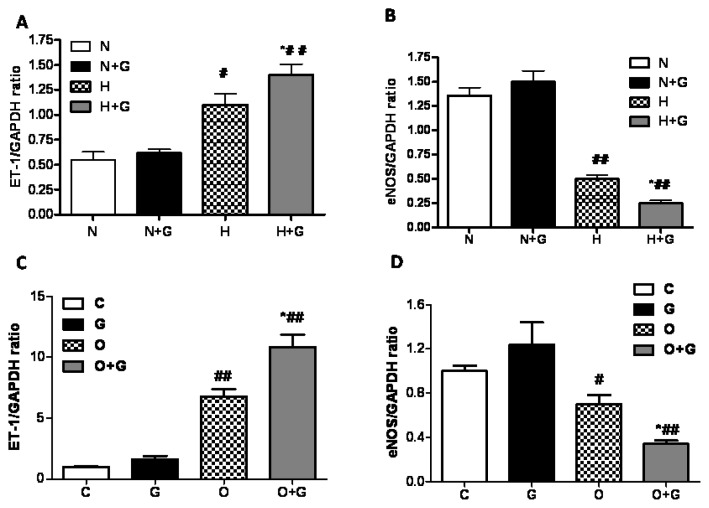
The effect of G-CSF on the expression of ET-1 and eNOS in thoracic aortae and endothelial cells. Analysis of ET-1 (**A**) and eNOS (**B**) mRNA expression in thoracic aortae. N: normal diet group, N + G: normal diet group treated with G-CSF, H: high-fat diet group. H + G. high-fat diet group treated with G-CSF. Results are expressed as means ± SEM. *p < 0.05, normal diet +G-CSF *vs.* normal diet group, or high-fat diet +G-CSF *vs.* high-fat diet group ^#^*p* < 0.05 ^##^*p* < 0.01 high-fat diet group *vs.* normal diet group or high-fat diet +G-CSF *vs.* normal diet +G-CSF. Analysis of ET-1 (**C**) and eNOS (**D**) mRNA levels in cultured endothelial cells. C: Control group. G: G-CSF group. O: OX-LDL group. O + G: OX-LDL + G-CSF group. Results are expressed as means ± SEM. *p < 0.05, G-CSF *vs.* control group, or OX-LDL +G-CSF *vs.* OX-LDL group ^#^*p* < 0.05, ^##^*p* < 0.01 OX-LDL *vs.* control group or OX-LDL + G-CSF *vs.* G-CSF group. Each experiment was repeated independently at least three times.

A previous study reported that ET-1 exacerbated endothelial dysfunction [17] and that endothelial nitric oxide synthase (eNOS) could exert vascular protective effects by producing nitric oxide (NO) [18]. We detected the expression of ET-1 and eNOS in thoracic aortae and cultured endothelial cells by the real-time PCR method. G-CSF treatment had no statistically significant effect on the ET-1 and eNOS expression in the thoracic aorta in rabbits fed a normal diet by real-time PCR analysis. High-fat diet induced an increase of ET-1 expression and a decrease of eNOS in the thoracic aorta (Figure 5A,B). In the high-fat diet group treated with G-CSF, ET-1 expression was further increased and eNOS expression was further decreased (Figure 5A,B). We next examined the mRNA expression of ET-1 and eNOS in cultured ECs incubated in OX-LDL for 24 h with or without G-CSF pretreatment. The results showed G-CSF promoted the increase of ET-1 expression and the decrease of eNOS expression in ECs with OX-LDL treatment but it did not affect the expression of ET-1 and eNOS in ECs with vehicle treatment (Figure 5C,D). It was consistent with the result *in vivo.* As an index for the impaired endothelial function, ET-1 exacerbates endothelial dysfunction to some extent [17]. Endothelial nitric oxide synthase (eNOS) produces nitric oxide (NO) from the amino acid L-arginine to exert vascular protective effects [18]. So G-CSF increased ET-1 expression and decreased eNOS expression in the hyperlipidemia state to impair endothelial dysfunction.

Thus, our data revealed that G-CSF deteriorated atherosclerosis by promoting lipid abnormity at the early phase and endothelial cell dysfunction by the ET-1/eNOS system in high-fat diet rabbits. In most clinical trials, five to six total doses of G-CSF were given over a one-week period. G-CSF may be a risk factor for exacerbating atherosclerosis especially in patients with hyperlipidemia. The doses of G-CSF used in the present study were similar to those used in humans. However, the dosing schedule was more frequent in that the animals received a total of 15 doses over a three-week period. Significant effects of G-CSF on the atherosclerotic lesion area were seen only in animals that also received a high-fat diet. It suggests that the increased course of G-CSF treatment may produce a deleterious effect on atherosclerosis especially in patients with the hyperlipidemia state. Our limitation is that we neither compared the effect of G-CSF on atherosclerosis at different frequencies nor explored other potential mechanisms. Randomized clinical trials to evaluate the feasibility and safety of G-CSF on coronary artery diseases induced by hyperlipidemia are warranted.

## 3. Materials and Methods

### 3.1. Animals and G-CSF Treatment

The 6-month-old male New Zealand white rabbits (Department of Experimental Animals, Chinese Academy of Sciences, 2–2.5 kg ) were randomized into four groups: normal diet plus vehicle group (*n* = 6); normal diet plus G-CSF group (*n* = 8); high-fat diet plus vehicle group (*n* = 8) and high-fat diet plus G-CSF group (*n* = 8). The normal diet groups were fed normal rabbit chow, whereas the high-fat diet rabbits were fed a diet containing 1% cholesterol and 5% lard for 10 weeks. In G-CSF-treated groups, recombinant human G-CSF (rhG-CSF, Kirin Pharma Co., LTD., Tokyo, Japan) was injected subcutaneously for the first 3 weeks (15 μg/kg/day, daily for the first 5 days per week). The same volume of saline was injected subcutaneously as control. All protocols were approved by the Institutional Animal Care and Use Committee of Fudan University, and in compliance with “Guidelines for the Care and Use of Laboratory Animals” published by the National Academy Press [19]. Six rabbits died in the course of the experiment: one in normal diet plus vehicle group, two in normal diet with G-CSF treatment group, two in high-fat diet with vehicle group, one in high-fat diet plus G-CSF group.

### 3.2. Plasma Lipid Content Analysis and White Blood Cell Counts

Total cholesterol (TC), triglyceride (TG), low density lipoprotein-cholesterol (LDL-C) and high cholesterol density lipoprotein-cholesterol (HDL-C) and the values of circulating white blood cells (WBC) were measured by the standard method in the Department of Laboratories in Zhongshan Hospital, Fudan University.

### 3.3. Morphological Analysis

The animals were sacrificed at the end of study and the thoracic aorta was excised for morphological analysis as follows: (1) Sudan III staining: the thoracic aortae cut to less than 2 cm were fixed by normal 10% neutral formalin and stained by Sudan III. The degree of atherosclerosis was evaluated as the percent of lesion area of the total surface area of the intima (the surface area of lesions/the surface area of the whole intima) with the Leica Qwin V3 analysis system. (2) Paraffin-embedded thoracic aorta tissues were subjected to hematoxylin-eosin (HE) staining by the standard procedure. (3) For scanning electron microscopy examination, thoracic aorta tissues were fixed in 2.5% glutaraldehyde, post-fixed for 1 h in 1% osmium tetroxide, rinsed with distilled water, dehydrated and then observed under a HITACHIS-520 scanning electron microscope. (4) Transmission electron microscopy analysis: thoracic aorta sections were fixed in 4% paraformaldehyde, post-fixed in 1% osmium tetroxide solution, dehydrated and embedded in epoxy resin. Semi-thin (1 μm) serial sections were prepared and ultra-thin sections (50 to 70 nm) were mounted on Formvar-coated (1595 E, Merck, Amsterdam, the Netherlands) 75 mesh copper grids and counterstained with uranyl acetate and lead citrate before analysis under a JOEL-1200 transmission electron microscope.

### 3.4. Cell Culture

Aortic endothelial cells were isolated from the thoracic aortae of rabbits by a previously described method [20]. The endothelial cells were cultured in High-glucose Dulbecco’s modified Eagle’s medium (DMEM) (Gibco, Langley, OK, USA) containing 20% fetal bovine serum (FBS, Hyclone, Logan, UT, USA) and seeded on a 6-well plate at 5 × 10^5^/well. These cells were pretreated with G-CSF (50 ng/mL) or vehicle for 30 min followed by stimulation with or without oxidized low density lipoprotein (OX-LDL, 50 μg/mL) for 24 h. Finally, these cells were collected for further TUNEL analysis and real-time RT-PCR analysis.

### 3.5. Apoptotic Cell Analysis by TUNEL Labeling

TUNEL labeling was performed according to the manufacturer’s protocol (*in situ* cell death detection kit. Merck Inc., CA, USA). The cells, grown on glass coverslips, were incubated with 50 μL TUNEL reaction mixture containing TdT for 60 min at 37 °C. DAB substrate solution was then added, and the mixture was incubated for 10 min at room temperature. The apoptotic cells were expressed as a percentage of TUNEL positive cells of all the cells obtained from the slides.

### 3.6. Reversal Transcription Polymerase Chain Reaction (RT-PCR)

Total RNA was extracted from thoracic aorta tissues or cultured endothelial cells, purified and reverse transcribed to cDNA. Real-time RT-PCR was carried out with a real-time PCR Detection System (BioRad, Hercules, CA, USA). GAPDH was used as an internal control to normalize for RNA amounts. The primers used were as follows: GAPDH, upstream primer, 5′-CCACTTTGTGAAGCTCATTTCCT-3′, downstream primer, 5′-TCGTCCTCCTCTGGTGCTCT-3′; ET-1, upstream primer, 5′-CTCTCTGCTGTTGGTGGCTTT-3′, downstream primer, 5′-TGGGTTTCCGCTCCTGT-3′; eNOS, upstream primer, 5′-AGGCCTCCTGTGAGACTTTC-3′, downstream primer, 5′-AAGGAGTCGAGGACTGGATG-3′.

### 3.7. Statistical Analysis

Data are expressed as mean ± SEM. The comparisons among groups were tested by one-way ANOVA. *p*-values < 0.05 were considered statistically significant. Statistical analyses were performed by the SPSS 13.0 software package (SPSS, Inc., Chicago, IL, USA).

## 4. Conclusions

Our results suggest that G-CSF and hyperlipidemia exert synergistic effects on lipid abnormity and endothelial cell dysfunction, which resulted in the deterioration of atherosclerosis in high-fat diet rabbits. The ET-1/eNOS system may regulate the progression. Therefore G-CSF may be a risk factor for exacerbating atherosclerosis especially in hyperlipidemic patients.
